# Forensic age estimation of the knee by post-mortem DR, CT, and MR imaging: a comparative study

**DOI:** 10.1007/s00414-024-03158-7

**Published:** 2024-01-19

**Authors:** Apameh Khatam-Lashgari, Mette Lønstrup Harving, Chiara Villa, Niels Lynnerup, Sara Tangmose Larsen

**Affiliations:** 1 Department of Forensic Medicine, Section of Forensic Pathology, University of Copenhagen, Copenhagen, Denmark; 2 Department of Radiology, Rigshospitalet, Copenhagen, Denmark

**Keywords:** Forensic age estimation, Ossification, Post-mortem, Imaging methods

## Abstract

It is believed by many that reference data for age estimation purposes must be imaging-modality specific. A study from our department has however proven otherwise. We therefore found it interesting to investigate this further by looking at the level of agreement between different imaging modalities. The aim of this study was to investigate the level of agreement between the three radiological modalities, computed tomography (CT), magnetic resonance imaging (MRI), and digital radiography (DR), in assessing the ossification of the epiphyses of the knee. A total of 34 deceased individuals of 10–25 years of age, brought in for a medicolegal autopsy at our department, were scanned by CT, MRI, and DR. The ossification stages of the three bones of the right knee, distal femoral, proximal tibial, and proximal fibular epiphysis were assessed using the established combined staging method by Schmeling et al. and Kellinghaus et al. Analysis of the results by Cohen’s weighted kappa showed a good agreement between CT and DR (*K* = 0.61–0.70), and MRI and DR (*K* = 0.68–0.79) but only moderate agreement between CT and MRI (*K* = 0.55–0.57). This leads us to conclude that different radiological images cannot be used interchangeably for age estimation purposes, so reference material needs to be imaging-modality specific. However, to make a more general conclusion research on a larger population is needed.

## Introduction

The growing global migration flows have led to increasing requests for age estimation of adolescents and young adults, as countries seek to adhere to the United Nations (UN) Convention on the Rights of the Child, which among other things specifies that refugee children must be treated the same way as other children in the country they are seeking refuge [1]. This has in turn led to a focus on the methods applied in forensic age estimation [2]. Although there is no consensus as to how to conduct forensic age estimation, many European countries follow the recommendations of the Study Group on Forensic Age Diagnostics (AGFAD) [3]. The current recommendations of this group involve a physical examination and radiographs of the left hand and the teeth [3]. In case the skeletal maturation of the bones of the hand is completed, assessment of the clavicle ossification is recommended by either conventional radiography (CR) or computed tomography (CT) [3].

To overcome concerns regarding radiation exposure of young individuals and to increase the accuracy of the currently recommended methods, interest has turned towards radiation-free imaging methods, such as magnetic resonance imaging (MRI) and ultrasound (US). In addition, in some countries, a specific medical indication is required to carry out X-rays on humans, which adds to the necessity of using alternative radiation-free imaging methods [4]. Although both MRI and US have been studied for diverse bones and several studies have been published in recent years, these non-invasive methods have yet to be included in the recommendations of AGFAD [5–16]. This may be due to a lack of reference materials and datasets meeting the groups’ criteria and a lack of evidence on the validity [3]. Many of the available MRI studies have shortcomings, and as a result, the only country that has implemented MRI in its routine age assessment has faced some criticism due to lack of proper validation, aside also criticism of the approach whereby a probable age is calculated [17]. We here focus on the image modality, rather than the latter point, although we may add that in Denmark [18], a minimum and probable age is reported as recommended by AGFAD [3].

The degree of ossification of the epiphyses of the knee is one of the anatomical markers that has shown potential for MRI use in forensic age estimation [5, 6, 19–24]. Using the knee also allows the use of extremity MRI scanners, which are considerably smaller and easier to use than whole-body MRI scanners [25]. Furthermore, motion artifacts are not a problem as is the case with the clavicle [26].

While MRI potentially is an excellent choice as a radiation-free alternative, its use for forensic age estimation has been questioned, as it is an expensive and time-consuming procedure that can be potentially stressful for the already traumatized asylum seeker [27], especially if carried out in a whole-body MRI scanner. Also, it must be considered that potential metal fragments might pose a problem, as asylum seekers often come from war zones [28]. Importantly, implementing MRI for age estimation needs building reference samples, as exist for radiographic methods. This ideally involves MRI of young living subjects of known age, and preferably of diverse ethnic and socio-economic background, as the reference datasets are applied to persons of uncertain provenance and socio-economical circumstance.

Several have argued that reference data should be imaging-modality specific [29–31]. However, one study has demonstrated good agreement between the ossification stages of the medial epiphysis of the clavicle on post-mortem CT (PMCT) and MRI (PMMRI) [32]. It is therefore interesting to explore if there is in fact good agreement between different imaging modalities regarding epiphyseal growth staging. We had the opportunity to investigate this at the Department of Forensic Medicine in Copenhagen since we have CT, MRI, and digital radiography (DR) at our disposal.

To date, there is no study comparing the developmental stages of the knee by CT, MRI, and DR. The aim of this study was, therefore, to compare the level of agreement between CT, MRI, and DR, for assessing the ossification of the epiphyses of the knee using the combined classification described by Schmeling et al. [33] and Kellinghaus et al. [34] on a deceased population of adolescents and young adults.

## Materials and methods

This prospective study included all autopsy cases in the age range of 10–25 years performed at the Department of Forensic Medicine at the University of Copenhagen from June 2020 to October 2022. PMCT, PMMRI, and PMDR images were conducted in connection with routine investigation, prior to the medico-legal autopsy. Exclusion criteria were severe decomposition, severe trauma near the growth plate, metal objects (e.g., intraosseous access or surgical fixation) in the knee, and the leg positioned in a way that would affect the staging process. If bone abnormalities were seen during the staging process, those cases were also excluded. A total of six were excluded from the study. In total, 34 subjects were included, nine females and 25 males (mean age females 21,67, mean age males 21,36) (Table 1).

**Table 1:** Age distribution for the subjects included in the study

Age (years)	Subjects (*n*)
10–15	3
16–18	4
> 18	27
Total	34

### Imaging modalities

MRI scans of the right knee were performed on a 1.5T Siemens Essenza scanner (Siemens Healthcare, Erlangen, Germany) using a knee coil (eight-channel extremity matrix coil, Siemens Healthcare, Erlangen, Germany). For the assessment of stages, T1-weighted turbo spin echo (T1-TSE) sequences in coronal and sagittal planes were used. The technical specifications were as follows: T1-TSE sagittal: TE 9.8 ms, TR 649 ms, FOV: 160 × 160 mm; scan time 4.48 min; flip angle: 150; matrix 218 × 256; slice thickness: 3 mm. T1-TSE coronal: TE 11 ms, TR 558 ms, FOV: 160 × 160 mm, scan time 3.41 min, flip angle 150, matrix 205 × 256, slice thickness 3 mm.

The CT scans were performed on a Siemens Somatom Definition AS (Siemens Healthcare, Erlangen, Germany). The CT scanning settings were as follows: kV 120/140 (depending on the size of the individual in the scanner), quality reference mAs 300, FOV 500 mm, slice thickness 2 mm. The images were analyzed in the coronal and sagittal plane using multiplanar reconstruction (MPR) and the bone window. 

Anterior/posterior (AP) and lateral radiographs of the right leg were performed using an X-ray machine Dragon X (Canon) digital scanner. All images were assessed using the Myrian software (Intrasense, Montpellier, France) [35].

### Image assessment

The ossification stages of the distal femoral epiphysis, proximal tibial epiphysis, and proximal fibular epiphysis were assessed using the combined classification system proposed and described by Schmeling et al. [33] and Kellinghaus et al. [34] (Table 2). The images were assessed in blind trials by AKL, a medical doctor with approximately two years of experience in forensic pathology including interpretation of whole-body PMCT. AKL reassessed the stages after a period of 2 weeks to 5 months for intraobserver agreement. To examine for interobserver agreement, all the images were assessed by MLH, a clinical radiologist with sub-specialization in musculoskeletal and trauma radiology.

**Table 2:** Combined classification system for assessment of ossification stages as described by Schmeling et al. [33] and Kellinghaus et al. [34]

Stage	Description
1	The ossification center has not yet ossified
2	The ossification center has ossified, but the epiphyseal cartilage has not ossified
2a	The lengthwise epiphyseal measurement is one-third or less compared to the widthwise measurement of the metaphyseal ending
2b	The lengthwise epiphyseal measurement is between one-third and two-thirds compared to the widthwise measurement of the metaphyseal ending
2c	The lengthwise epiphyseal measurement is over two-thirds compared to the widthwise measurement of the metaphyseal ending
3	The epiphyseal cartilage is partially ossified
3a	The epiphyseal-metaphyseal fusion completes one-third or less of the former gap between epiphysis and metaphysis
3b	The epiphyseal-metaphyseal fusion completes between one-third and two-thirds of the former gap between epiphysis and metaphysis
3c	The epiphyseal-metaphyseal fusion completes over two-thirds of the former gap between epiphysis and metaphysis
4	The epiphyseal cartilage is fully ossified and the epiphyseal scar is visible
5	The epiphyseal cartilage has fused completely and the epiphyseal scar is no longer visible

The PMDR images were assessed in both AP and lateral projection, and all the PMMRI and PMCT slices were assessed in both coronal and sagittal plane before an overall stage was assigned. In cases of disagreement between the two staging grades (coronal/sagittal plane or AP/lateral projection), the lowest stage given was applied.

To ensure consistency in the assigning of the stages, the observers agreed on some guidelines for assessing PMCT and PMMRI images. For stages 2a–c and 3a–c, the stage that appeared on most slices was decisive. A single tiny gap in one slice was not enough to assign stage 3c, and those cases were assessed as having fully ossified and were assigned stage 4. On PMCT images, stage 5 was assigned when the epiphyseal line was no longer visible centrally and was indistinct at the edges.

### Statistical analysis

Data were analyzed using the SPSS (Statistical Package for Social Sciences) version 28 for Windows. Cohen’s weighted Kappa with quadratic weights was run to analyze intraobserver and interobserver agreement as well as the agreement between modalities. The strength of the agreement was classified according to Altman [36]. Bland Altman plots were produced for a graphical visualization of the agreement of grading between the modalities.

## Results

**Fig. 1:**
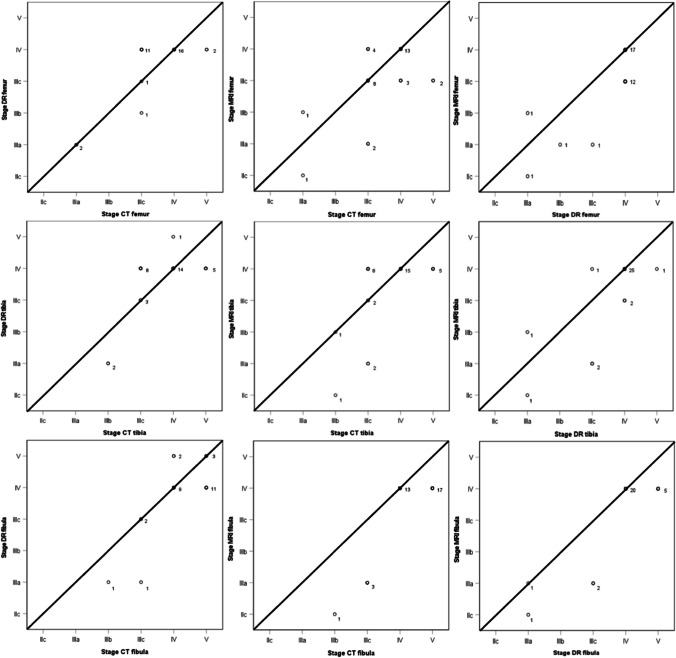
Scatter plots of agreement between stages assessed using PMCT, PMDR, and PMMRI for the femoral bone, the tibial bone, and the fibular bone. The number next to the data point indicates the number of subjects

**Fig. 2:**
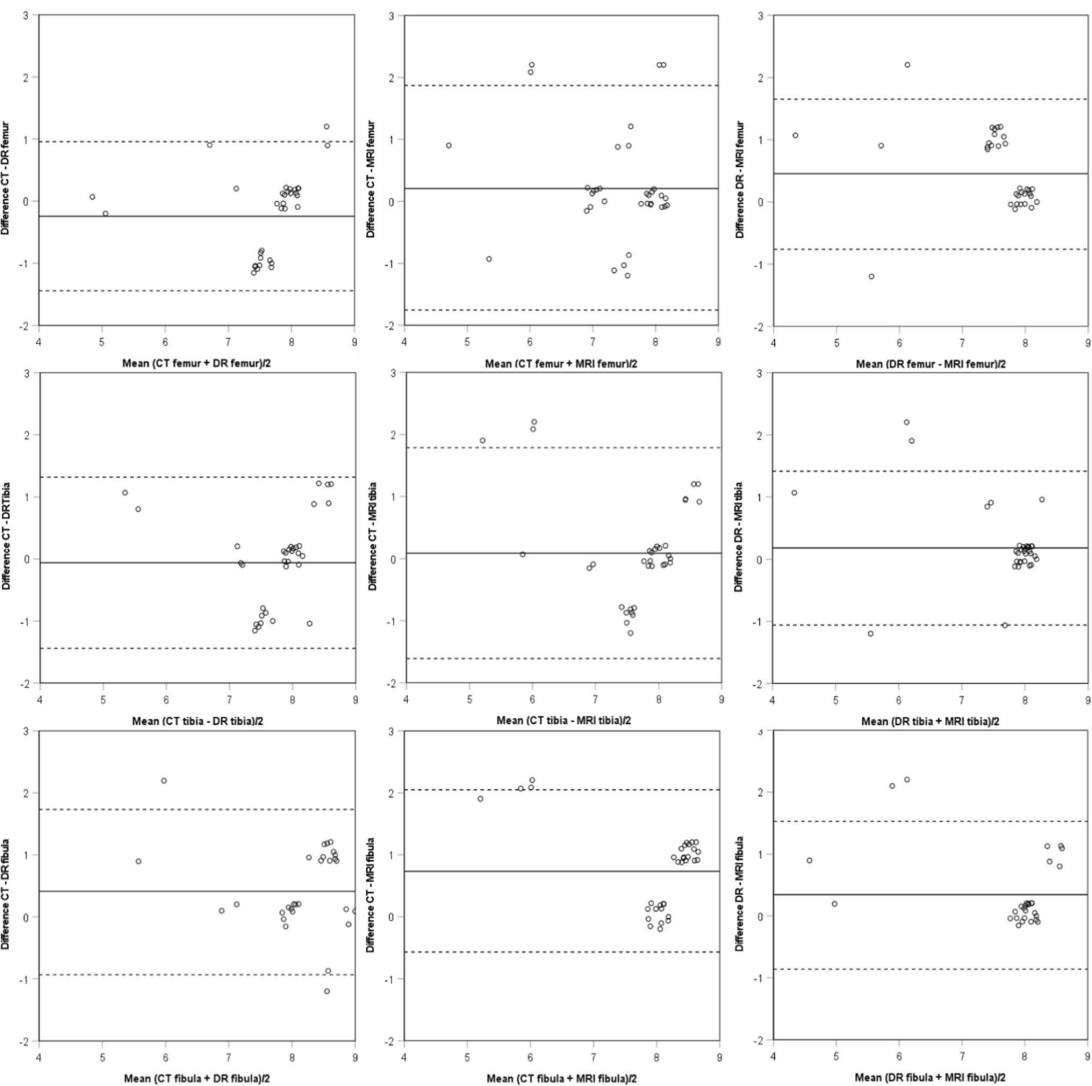
Bland Altman plots showing the difference against the mean of the stages for PMCT, PMDR, and PMMRI and the limits of agreement (dash lines). To obtain these plots, the stages were given a consecutive number; thus, stage 1 was assigned 1, stage 2a was assigned 2, stage 2b was assigned 3, stage 2c was assigned 4, stage 3a was assigned 5, stage 3b was assigned 6, stage 3c was assigned 7, stage 4 was assigned 8, and stages 5 was assigned 9. Jittering was applied for overlapping data points, which affects the exact value of the data points

**Fig. 3:**
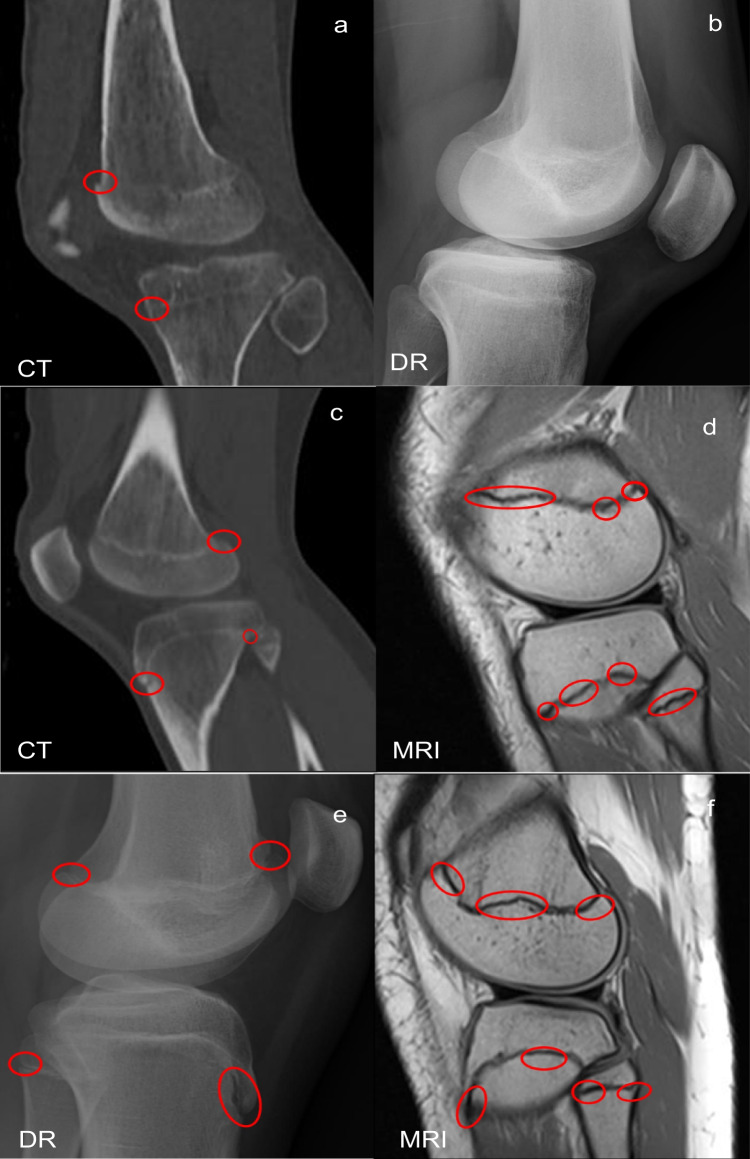
Examples of images with disagreement between modalities for PMCT vs PMDR (**a** and **b**), PMCT vs PMMRI (**c** and **d**), and PMDR vs PMMRI (**e** and **f**). In the case of PMCT vs PMDR, the femoral and tibial bone were staged 3c and the fibular bone was staged 4 on the PMCT image (**a**) while stage 4 was assigned on the PMDR image (**b**) for all three bones. In the case of PMCT vs PMMRI, stage 3c was assigned on the PMCT image (**c**), while stage 3a was assigned on the PMMRI image (**d**) for all three bones. In the case of PMDR vs PMMRI, stage 3c was assigned on the PMDR image (**e**), while stage 3a was assigned on the PMMRI image (**f**) for all three bones. The marked areas (red circles) represent the parts of the epiphyseal plates that have not fused. The PMCT slice of the proximal fibular epiphysis seen in image **c** does not depict the stage given as it was impossible to find a suitable slice depicting all three bones showing the stage given

Descriptive statistics for each stage stratified by sex and modalities are given in Table 3. It was not possible to assess the ossification stages of the distal femoral, proximal tibial, and proximal fibular epiphysis in all the cases included. In four of the 34 cases, staging of the fibular bone on PMDR was not possible due to substantial superimposition by the tibial bone either in AP or lateral projection, and in one case, the lateral projection was missing leading to a complete exclusion of the PMDR results for that case. Overall, the mean chronological age increases with increasing stages for all three bones and all three modalities. However, it must be noted that all the stages observed are not represented for each modality due to the low number of cases included. Table 4 presents an overview of the distribution of the assigned stages for the ages 10–15 years, 16–18 years, and above 18 years. A comparison of the three modalities using kappa statistics revealed good agreement between stages assessed by PMCT and PMDR and by PMMRI and PMDR but only moderate agreement between stages assessed by PMCT and PMMRI for all three bones (Table 5). There was good to very good intraobserver and interobserver agreement, except for the interobserver agreement of PMCT tibia in which case it was only fair (Table 5). Intraobserver and interobserver agreement was overall higher for PMMRI and PMDR compared to PMCT. Comparing all three modalities, we found that there was complete agreement in 13/33, 14/33, and 9/29 cases for the femoral, tibial, and fibular bone, respectively. To obtain a graphical visualization of the agreement between the modalities, data for all three bones as well as the “line of equality” were plotted (Fig. 1). Generally, there was a tendency that a higher stage was given on PMDR images as compared to both PMMRI (for all three bones) and PMCT (femoral and tibial bone). Although only seen for the femoral and fibular bone, there was a tendency that a higher stage was given on PMCT images as compared to PMMRI. These findings are further confirmed on the Bland Altmann plots of the difference against the mean of the stages (Fig. 2). The mean difference and standard deviation for the plots can be seen in Table 6. As seen in Table 7, 8, and 9, in most cases, it is a matter of one-stage disagreement, and in a few cases, the disagreement is by two stages. The disagreement by two stages is primarily seen in PMCT vs PMMRI which may have affected the lower kappa values between these modalities. In addition, it seems that the disagreement does not depend on the bone but rather on the modalities. Though it must be noted that substages have been accounted for as a complete stage in the transformation. In all but three of the cases with a two-stage difference, the disagreement was within the substages 3a–3c. Image examples of disagreement between stages are represented in Fig. 3.

**Table 3:** Mean (± standard deviation) and minimum and maximum ages in years for ossification stages of the distal femoral epiphysis, proximal tibial epiphysis, and proximal fibular epiphysis on PMCT, PMDR, and PMMRI. Data with *n* < 3 is not shown

Stage	Sex	Femur						Tibia						Fibula					
		PMCT		PMDR		PMMRI		PMCT		PMDR		PMMRI		PMCT		PMDR		PMMRI	
		*N*	Mean ± SD (min–max)	N	Mean ± SD (min–max)	N	Mean ± SD (min–max)	*N*	Mean ± SD (min–max)	*N*	Mean ± SD (min–max)	*N*	Mean ± SD (min–max)	*N*	Mean ± SD (min–max)	*N*	Mean ± SD (min–max)	*N*	Mean ± SD (min–max)
**3c**	Female	3	20.33 ± 5.51 (14–24)	-	-	-	-	4	21.50 ± 5.10 (14–25)	-	-	-	-	-	-	-	-	-	-
	Male	11	20.81 ± 2.96 (16–25)	-	-	13	21.92 ± 2.66 (16–25)	8	20.25 ± 3.33 (16–25)	-	-	-	-	-	-	-	-	-	-
**4**	Female	6	22.33 ± 3.27 (16–25)	8	22.63 ± 2.83 (16–25)	8	22.63 ± 2.83 (16–25)	-	-	8	22.63 ± 2.83 (16–25)	8	22.63 ± 2.83 (16–25)	-	-	6	22.33 ± 3.27 (16–25)	8	22.63 ± 2.83 (16–25)
	Male	10	23.30 ± 1.70 (21–25)	21	22.62 ± 2.36 (16–25)	9	23.33 ± 1.66 (21–25)	13	23.08 ± 1.55 (21–25)	19	22.74 ± 2.16 (16–25)	20	22.50 ± 2.48 (16–25)	12	21.58 ± 2.71 (16–25)	14	22.21 ± 2.55 (16–25)	22	22.50 ± 2.37 (16–25)
**5**	Female	-	-	-	-	-	-	3	20.67 ± 4.16 (16–24)	-	-	-	-	7	22.57 ± 3.05 (16–25)	-	-	-	-
	Male	-	-	-	-	-	-	-	-	-	-	-	-	10	23.60 ± 1.27 (22–25)	3	22.33 ± 2.31 (21–25)	-	-

**Table 4:** Overview of stages assigned for a given chronological age span for both females and males for each imaging modality and bone

Chronological age (years)	Stage assigned								
	PMCT			PMDR			PMMRI		
	Femur	Tibia	Fibula	Femur	Tibia	Fibula	Femur	Tibia	Fibula
10–15	3a–3c	3b–3c	3b–3c	3a–3c	3a–3c	3a–3c	2c–3b	2c–3b	2c–3a
16–18	3c–4	3c–5	3c–5	3b–4	3c–4	3c–4	3a–4	3a–4	3a–4
> 18	3c–5	3c–5	4–5	4	4–5	4–5	3c–4	3c–4	4

**Table 5:** Cohen’s weighted Kappa coefficient for intra- and interobserver agreement as well as agreement between modalities

	Intraobserver agreement			Interobserver agreement			Agreement between modalities		
	PMCT	PMDR	PMMRI	PMCT	PMDR	PMMRI	PMCT vs PMDR	PMCT vs PMMRI	PMDR vs PMMRI
Femur	0.801	0.973	0.934	0.797	0.882	0.836	0.700	0.573	0.683
Tibia	0.718	0.911	0.879	0.404	0.652	0.839	0.611	0.553	0.746
Fibula	0.822	0.902	0.919	0.648	0.633	0.943	0.624	0.567	0.786

**Table 6:** Mean difference and standard deviation for the Bland Altman plots

	PMCT vs PMDR		PMCT vs PMMRI		PMDR vs PMMRI	
	Mean	SD	Mean	SD	Mean	SD
Femur	− 0.24	0.61	0.21	0.85	0.45	0.62
Tibia	− 0.06	0.70	0.09	0.87	0.18	0.64
Fibula	0.41	0.68	0.74	0.67	0.34	0.61

**Table 7:** Overview of the level of agreement for all the cases of the femoral bone

Femur	PMCT vs PMDR	PMCT vs PMMRI	PMDR vs PMMRI
Complete agreement	19/33 (58%)	21/34 (62%)	17/33 (52%)
Disagreement by one stage	14/33 (42%)	9/34 (26%)	15/33 (45%)
Disagreement by two stages	-	4/34 (12%)	1/33 (3%)

**Table 8:** Overview of the level of agreement for all the cases of the tibial bone

Tibia	PMCT vs PMDR	PMCT vs PMMRI	PMDR vs PMMRI
Complete agreement	17/33 (52%)	18/34 (53%)	25/33 (76%)
Disagreement by one stage	16/33 (48%)	13/34 (38%)	6/33 (18%)
Disagreement by two stages	-	3/34 (9%)	2/33 (6%)

**Table 9:** Overview of the level of agreement for all the cases of the fibular bone

Fibula	PMCT vs PMDR	PMCT vs PMMRI	PMDR vs PMMRI
Complete agreement	14/29 (48.3%)	13/34 (38%)	21/29 (72%)
Disagreement by one stage	14/29 (48.3%)	17/34 (50%)	6/29 (21%)
Disagreement by two stages	1/29 (3.4%)	4/34 (12%)	2/29 (7%)

## Discussion

In this study, we investigated the application of the three modalities CT, DR, and MRI for age assessment of the knee in the same individual. A comparison of the three modalities reveals that there is good agreement between PMDR and PMCT as well as PMDR and PMMRI, but only moderate agreement between PMCT and PMMRI. Few have done comparative studies dealing with forensic age estimation [13, 29–32, 37]. Therefore, a comparison of our results to other studies is limited. Nevertheless, this study can shed light on the ossification stages across different modalities and provide additional insight into post-mortem imaging.

The fact that there is merely moderate agreement between PMCT and PMMRI images is not in line with a previous study conducted in our department which concluded that CT and MRI of the clavicles used for forensic age estimation purposes can be used interchangeably [32]. However, this study was on a different bone; it used fewer stages and included only one growth plate, which may result in a higher level of agreement. On the other hand, this partially verifies what other authors have previously claimed, namely, that modality-specific reference data is necessary in the practice of age estimation [29–31]. This is further confirmed by the fact that complete agreement for all three modalities was only seen in 13/33, 14/33, and 9/29 cases for the femoral, tibial, and fibular bone, respectively. Overall we observed a higher stage for PMDR compared to PMCT and PMMRI, respectively, which is similar to other studies [20, 29, 30, 37, 38]. Moreover, similar to other studies, we found that epiphyseal fusion/closure occurs earlier on DR images than MRI images [20, 37] and specifically also for the distal femoral epiphysis in males [37]. This may be due to the better contrast and definition provided by MRI [20]. In our study, stage 5 was not observed on PMMRI images but only on PMCT and PMDR images. This is in line with other studies that have applied the staging method by Schmeling et al. and Kellinghaus et al. and with a similar MRI protocol as ours [5, 6, 19, 39]. In those studies, they conclude that the reason must be that stage 5 (disappearance of the epiphyseal scar) lies above their included age limit or the possibility that the epiphyseal scar persists into old age. In our study, stage 5 was observed on several PMCT and PMDR images, whereas it was not observed on PMMRI. This observation indicates that the above-mentioned qualities of the MRI might explain the missing stage 5 in our study and those earlier studies.

The lower intraobserver and interobserver agreement seen for PMCT compared to the other two modalities may be ascribed to the slice thickness. In four cases found in our archive, both 1.0 mm and 2.0 mm PMCT images were available and a comparison of those showed that in more than 50% of the cases, a lower stage was given on 1.0 mm CT images compared to 2.0 mm. This indicates that slice thickness is just as important for the staging of the knee on CT images as it is for the clavicles. The slice thickness was 2 mm in our study, and 1 mm is recommended for the staging of clavicles on CT images [40]. We also noted that our interobserver agreement was not as good as our intraobserver agreement which might be due to the different experience and the qualification of the observers. It has been shown that experience and specific qualification affects the epiphyseal staging of clavicles [41]. In addition, interobserver agreement was generally higher for the distal femoral epiphysis compared to the proximal tibial epiphysis. This has previously been noticed and explained by the thicker shape of the femur compared to the tibia [20].

When comparing our results with other studies using the same staging method and MRI sequence, we found a minimum age of 16 years for stage 3c for the distal femoral epiphysis in males which is similar to the minimum age of 16.13 years obtained by Ottow et al. [19] and 15.8 years obtained by Ekizoglu et al. [39]. For females, the minimum age for stage 4 for the distal femoral epiphysis and proximal tibial epiphysis was 16 years which is similar to the minimum ages 16.2 and 15.6 and 16.13 and 15.87 years obtained by Krämer et al. [5, 6] and Ottow et al. [19], respectively. In our study, the minimum age obtained for stage 4 for the proximal tibial epiphysis was 16 years for males, which compares to the minimum ages 16.3, 15.90, and 15.8 years from the studies of Krämer et al. [5], Fan et al. [37]. and Ekizoglu et al. [39], respectively. However, our minimum age of 21 years for males for stage 4 for the distal femoral epiphysis is much higher than the results from the other studies [6, 19, 37, 39]. This result suggests that stage 4 of the distal femoral epiphysis may be helpful in the estimation of the 18-year age limit as previously proposed by some authors [6, 21] but then disproved by others [19, 37, 39]. However, the validity of the minimum and maximum ages is limited by the low number of cases and the lack of even distribution in terms of age and sex. Indeed, due to the unbalanced age distribution with most of the subjects included above 18 years of age, selection bias needs to be considered. The question of the 18-year-old limit must be verified on a larger population with a more evenly age and sex distribution.

There are limitations to this study which need to be mentioned and considered. As mentioned in the previous paragraph, the low number of subjects included and the unbalanced distribution in both age and sex are evident limitations. Another possible limitation and probably the most important one in this context is the question of whether research on post-mortem individuals is transferable to living individuals. It is well-known that radiological images conducted post-mortem look differently compared to images of living individuals. For instance, MRI images are influenced by body temperature, and CT images by putrefaction [42, 43]. Nevertheless, a previous study comparing clavicle fusion in living and post-mortem individuals showed no statistically significant difference in stages, thereby concluding that post-mortem MRI (PMMRI) and MRI of the living are likely transferable [16]. A final limitation which needs to be mentioned is the fact that the scan protocol of the PMCT images was meant for our routine investigation and not the assessment of knee epiphysis. For instance, we observed a lower in-plane resolution when assessing the images in coronal and sagittal plane using MPR which could have been improved by reconstructing from isotropic voxels. Furthermore, we could have achieved a higher in-plane resolution on axial images by adjusting the FOV for our axial scan.

On the other hand, a strength of our study is that we have assessed the images in more than one projection or plane including all slices and all three bones of the knee, while many other studies involving epiphyseal growth staging of the knee have chosen a specific region of the bone to assess and only in one plane or projection [5, 6, 13, 19, 22, 23, 37, 39, 44, 45]. Though, some authors advise against the assessment of the proximal fibular epiphysis on MRI due to its lack of visibility in all slices and therefore might see its inclusion in this study as a limitation rather than a strength [19]. This methodological approach was applied in order to adhere to the benefit of the doubt principle. Assessing all planes/projections and all slices guarantees that uncertainties in the staging process can be accounted for. Nevertheless, assessment of all slices in more than one plane or projection could perhaps also explain the disagreement between observers and modalities since the more images there are to assess the more risk of disagreement/doubt on the stage to assign. 

Variations both across different bones and among different individuals make it difficult to find a method for forensic age estimation that is suitable for all individuals and in any case. Therefore, studies on age estimation differ in their choice of study setup including the staging method. The combined classification by Schmeling et al. and Kellinghaus et al. that was applied in this study was developed for the clavicles using CR and CT, respectively. Various studies have shown the reproducibility and feasibility of this staging method on CR, CT, and MRI images [5, 29, 41]. We, therefore, chose to apply this method in our study and believed it was the best choice for comparison of modalities than any of the other staging methods available.

## Conclusions

In conclusion, we found good agreement between PMDR and both PMCT and PMMRI but moderate agreement between PMCT and PMMRI. These results suggest that reference data for developmental stages of individual bones should be imaging-modality specific. However, research on a larger population is needed to validate our findings.

## Data Availability

The datasets generated during and/or analyzed during the current study are available from the corresponding author upon reasonable request.

## Declarations

### Data approval

The handling of data was approved by The University of Copenhagen on behalf of the Danish Data Protection Agency (514–0614/21–3000).
